# Laceration of the Perineum

**Published:** 1887-09

**Authors:** J. M. Sanders

**Affiliations:** Magnolia, S. C.


### LACERATION OF THE PERINEUM.

BY J. M. SANDERS, MAGNOLIA, S. C.

Laceration of the perineum during parturition is a misfortune much to be deplored. When primipara cases occur after the age of thirty, owing to the then more dense and unyielding state of the structure involved, it is sometimes impossible to prevent its rupture by the means usually advised to secure its integrity. What, then, is the cause of our failures? It seems that one main cause of non-success is that all our efforts in this line are never brought into operation at a time early enough to become available. When the time for action arrives, are we not conscious that we have lost an opportunity for the better preparation of the part for the ordeal through which it must inevitably and speedily pass? Was there never a time when this last hindrance to fatal exit might have been rendered more feeble and non-resisting? (Would an earlier marriage have been better?) I have witnessed lying-in cases of women marrying at thirty-five who suffered from perinael rent, which no resources of our art could have prevented, at the time I was called in, short of absolute butchery—so small was the degree of preparation or relaxation of the part beforehand. This is a humiliating confession.

When taking charge of a case of labor, we are advised (1) to allow all reasonable delay during the first stage, and this is a rule that should be followed in all labor cases unless peculiar and unusual circumstances compel non-observance. The object of this delay (or simply letting alone) is to give nature full time to carry on her relaxing processes. (2) Pressing back the fœtal head during the last throes of labor, where it is pressing hard against the perineum, so as to prevent its precipitate and violent exit through the unyielding part, is but a continuation of dilatory measures. We are still trusting to nature to relax her grip. (3) Manipulation through the rectal channel with the design of more favorably directing the head is another means adopted for guarding against personal rupture. (4) The making of incisions through the tense parts, other than at the fourchette, gives capacity, but this is substituting lacerations less formidable for one more so. But all these means sometimes fail, and the perineum, proving too stubborn to yield, must be rent asunder, leaving a ghastly wound, difficult to heal, excessively annoying and ready prepared for the introduction of septic material into the circulation.

What, then, can be done the better to prevent so grievous a calamity? We know of instances where certain means have been employed before *accouchement* in anticipation of certain troubles during and thereafter looked for. There is the treatment of counter-sunk nipple by suction, the toughening of its thin and tender cuticle by proper lavements. We also palliate the pains of distending abdominal walls before-time with anodyne and lubricating liniments, etc. Then why should a perineum be left to its fate when we know its peril? Would that some inventor of corsets, garters, abdominal supports, eugenias, conception preventors, etc., go to work and make an instrument to increase perinæl capacity which would prove satisfactory to all parties concerned.

